# Spray-dried cyclophosphamide-loaded polyhydroxyalkanoate microparticles: design and characterization

**DOI:** 10.5599/admet.2434

**Published:** 2024-10-09

**Authors:** Sergei Lipaikin, Aleksei Dorokhin, Galina Ryltseva, Andrey Oberenko, Evgeniy Kiselev, Alexander Shabanov, Tatiana Volova, Ekaterina Shishatskaya

**Affiliations:** 1 Siberian Federal University, 79 Svobodny pr., Krasnoyarsk 660041, Russia; 2 Institute of Biophysics SB RAS, Federal Research Center “Krasnoyarsk Science Center SB RAS”, 50/50 Akademgorodok, Krasnoyarsk 660036, Russia; 3 L.V. Kirensky Institute of Physics, Siberian Branch of the Russian Academy of Sciences, 50/12 Akademgorodok, Krasnoyarsk 660036, Russia

**Keywords:** Microencapsulation, drug loading, drug release

## Abstract

**Background and purpose:**

Cyclophosphamide (CP) is a widely used antitumor and immunosuppressive drug, but it is highly cytotoxic and has carcinogenic and teratogenic potential. To reduce adverse effects of CP therapy and the frequency of its administration, the microencapsulation of CP into biodegradable polymeric matrices can be performed. However, according to the literature, only a few polymers were found suitable to encapsulate CP and achieve its’ sustained release.

**Experimental approach:**

In this research, spray-dried cyclophosphamide-loaded poly(3-hydroxybutyrate-co-3-hydroxyvalerate) (PHBV) microparticles were prepared and characterized in terms of their average hydrodynamic diameter, polydispersity index, surface morphology, zeta potential, encapsulation efficiency, drug loading, thermal properties and cytotoxicity against 3T3 cells.

**Key results:**

The obtained CP-loaded microparticles had a regular spherical shape, uniform size distribution with an average diameter of 4.21±0.04 μm and zeta potential of -34.2±0.2 mV. The encapsulation of cyclophosphamide into the PHBV matrix led to a decrease in melting and degradation temperatures and an increase in diameter, glass transition and cold crystallization temperatures compared to blank microparticles. Moreover, microencapsulation of cyclophosphamide lowered its cytotoxicity compared to the pure drug: the number of dead cells in the culture decreased by 28 %, while their metabolic activity increased by 20 %. The cumulative *in vitro* drug release studies showed a gradual release of CP up to 18 days, so the obtained microparticle formulation can be used as a sustained-release cyclophosphamide delivery system.

**Conclusion:**

In this research, a novel cyclophosphamide-loaded platform based on PHBV microparticles was established and characterized. Overall, this study offers promising prospects for cancer therapy in the future.

## Introduction

Cyclophosphamide (CP) remains one of the most successful antineoplastic agents. Even today, since its synthesis in 1958 [1], cyclophosphamide is still widely used as a chemotherapeutic agent and as an immunosuppressive drug in blood and marrow transplantation. CP is used to treat various types of diseases like lymphoma, myeloma, sarcoma, breast cancer, ovarian cancer, leukaemia, *etc* [2-4]. The duration of cyclophosphamide therapy depends on the type and severity of illness and can range from several days to several months [5,6] or even years [7,8].

Nevertheless, CP is highly cytotoxic and like other alkylating agents, it is toxic predominantly to rapidly proliferating cells and tissues such as the haematopoietic system, hair follicles and so on. Well-known side toxic effects of conventional doses of cyclophosphamide are nausea, alopecia, infertility, pulmonary fibrosis, bladder injury, *etc* [9,10]. Furthermore, CP has carcinogenic and teratogenic potential [4,11].

To decrease the frequency of administration and the cytotoxic effects of cyclophosphamide and to achieve its controlled and sustained release, the microencapsulation of CP into the polymer matrices can be performed. There are known liposome-encapsulated [12], polylactic and poly-ε-caprolactone [13] microstructural carriers, poly(D,L-lactide-co-glycolide) [14,15] and chitosan [16] microspherical carriers of cyclophosphamide. Nevertheless, according to the literature, no studies have been conducted on the formation of polyhydroxyalkanoate microparticles containing cyclophosphamide.

Polyhydroxyalkanoates (PHAs) are a group of thermoplastic biopolymers of natural origin that are currently regarded as promising alternatives to synthetic, indestructible plastics [17,18]. PHAs are biocompatible, completely biodegradable and environmentally friendly materials: renewable sources (for example, waste fish oils [19], waste cooking oils [20], lignocellulose biomass waste [21], *etc*.) and greenhouse gases [22] are used as substrates for bacterial synthesis of PHAs [23]. The family of PHAs includes approximately 150 different types of monomers, which are synthesized by various microorganisms and differ significantly from each other in terms of their physico-chemical properties [21].

The aim of the research is to obtain and investigate the properties of poly(3-hydroxybutyrate-co-3-hydroxyvalerate) microparticles containing cyclophosphamide.

## Experimental

### Materials

Microbial poly(3-hydroxybutyrate-co-3-hydroxyvalerate) (*M*_w_ = 490.8 kDa, 10 mol.% of 3-hydroxyvalerate) was produced at the laboratory of Biotechnology of new biomaterials of Siberian Federal University, Russian Federation [24]. Cyclophosphamide monohydrate, dichloromethane (DCM), trichloromethane (TCM), dimethyl sulfoxide (DMSO), sulphamic acid and sodium nitrite were purchased from Merck (Germany).

Dulbecco’s Modified Eagle Medium (DMEM), 3-(4,5-dimethylthiazol-2-yl)-2,5-diphenyltetrazolium bromide (MTT) and ReadyProbes™ Cell Viability Imaging Kit were obtained from Thermo Scientific (USA). Fetal Bovine Serum (FBS) and antibiotic-antimycotic solution were purchased from Sigma-Aldrich (USA).

All reagents and solvents were of analytical grade and used as received without further purification. The water used was purified by Arium® Pro Ultrapure water system (Sartorius AG, Germany).

### Preparation of PHBV microparticles

PHBV microparticles (MPs) were prepared by spray drying of PHBV/DCM solution using Büchi Mini Spray Dryer B-290 (BUCHI Laboratory Equipment, Switzerland). The operating parameters were set as follows: argon gas flow rate 35 m^3^ h^-1^, PHBV/DCM solution 0.1 % (w/v), inlet temperature 100 °C, PHBV solution feed rate 7.5 mL min^-1^. The resulting MPs were collected, weighted and kept at -20 °C for further subsequent use in experiments.

Cyclophosphamide-loaded microparticles (CP-MPs) were obtained under similar conditions, except that the PHBV/DCM solution contained CP (CP to PHBV ratio 1:10).

### Yield of microparticles, particle size, size distribution and zeta potential

The total yield (*Y* / %) of microparticles was determined according to Equation (1):



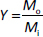

(1)


where *M*_o_ is the mass of the obtained microparticles and *M*_i_ is the total mass of PHBV and CP used in the synthesis.

To determine the average hydrodynamic particle diameter, polydispersity index (PDI) and zeta potential of the obtained microparticles, Zetasizer Nano ZS (Malvern, UK) was used. 0.3 mg of each sample was suspended in 2 mL of deionized water and sonicated at 30 W for 1 min before the measurements.

### Scanning electron microscopy

The morphology of the obtained microparticles was studied using Scanning Electron Microscope SU3500 (Hitachi, Japan). To obtain high-quality SEM images (by increasing conductivity and promoting heat dissipation from polymer matrix), microparticles were coated with 5 nm platinum layer using Leica EM ACE200 (Leica Microsystems, Germany).

### Determination of CP encapsulation efficiency and drug loading

The encapsulation efficiency (EE) and drug loading (DL) of CP were determined by ultraviolet-visible spectroscopy using Genesys 10S UV-Vis (Thermo Scientific, USA) according to the procedure described in [25]. Briefly, 10 mg of CP-MPs were dissolved in 1 mL of TCM, added to the solution containing 1 mL of water, 1 mL of 5 % (w/v) HCl, 1 mL of 20 % (w/v) NaNO_2_ and heated in a water bath at 60-65 °C for 20 min. The solution was cooled under tap water and mixed with 5 mL of 15 % sulphamic acid and 2 mL of 20 % (w/v) NaOH. The resultant solution was transferred into a volumetric flask and filled up to the mark with water. The absorbance was measured at 325.0 nm against the blank solution. The amount of CP in microparticles was determined using the calibration curve.

The encapsulation efficiency (EE, %) was calculated according to Equation (2):



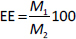

(2)


where *M*_1_ is the mass of CP in CP-MPs and *M*_2_ is the initial mass of CP.

Drug loading was calculated by Equation (3):



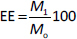

(3)


### Thermal properties

The glass transition temperature (*T*_g_), cold crystallization point (*T*_cc_), melting point (*T*_m_), and thermal degradation temperature (*T*_deg_) of the microparticles were determined by differential scanning calorimetry (DSC) and thermogravimetric analysis (TGA). DSC analysis was carried out using a DSC-1 differential scanning calorimeter (Mettler Toledo, Switzerland). 3-5 mg of each sample was placed in an aluminium crucible and heated up to 200 °C at a rate of 5 °C min^-1^, held at 200 °C for 1 min, then cooled to -20 °C (5 °C min^-1^) and held for 4 min. Each sample was further reheated to 200 °C at a rate of 5 °C min^-1^. TGA was performed using TGA 1 (Mettler Toledo, Switzerland). The samples (3 to 5 mg) were placed in a ceramic crucible and heated from 50 to 600 °C at a rate of 10 °C min^-1^. DSC and TGA analyses were performed under a nitrogen atmosphere. Thermograms were analysed using the “StarE” software.

### FTIR spectroscopy

Fourier transform infrared spectroscopy (FTIR) analysis of the obtained microparticles was performed using a FT-801 FTIR spectrometer ("SIMEKS", Russia). The samples were dissolved in TCM and left to evaporate the solvent to form a thin film. The FTIR spectra of the polymer films were recorded at 400 to 4000 cm^-1^ at room temperature.

To analyze the functional groups on the surface of the obtained microparticles ATR-FTIR spectroscopy technique was performed using ATR-accessory for FT-801 FTIR spectrometer (ZnSe crystal).

Data acquisition is performed at a resolution of 4 cm^-1^, employing 64 scans per spectrum.

### In vitro release

The investigation of the release of CP from CP-MPs was carried out *in vitro*. For this purpose, 2 mg of CP-MPs samples were suspended in 2 mL of phosphate-buffered saline (pH 7.4) in a 2.5 mL sterile centrifuge tubes. The tubes were thermostated at 37 °C. At predetermined time intervals, the solution was withdrawn from the tube, centrifuged at 11000 rpm for 5 min, and the supernatant was collected and successively treated with HCl, NaNO_2_, sulphamic acid and NaOH as mentioned above. The amount of CP released was determined by the measurement of the absorbance of the solution at 325.0 nm (using a calibration curve). All measurements were performed in triplicate. The percentage of CP released at each time point was calculated by dividing the data obtained at each time by the total amount of CP inside the microparticles.

### In vitro cell viability and cytotoxicity assays

*In vitro* cell viability of 3T3 fibroblast cells was estimated in the presence of CP, MPs and CP-MPs. The cells were cultivated in DMEM with the addition of FBS and antibiotic-antimycotic solution in СО_2_ incubator (Sanyo, Japan). 3T3 cells were incubated for 24 h with the initial concentration of 2·10^4^ cells cm^-2^. After incubation, the medium was replaced by a fresh culture medium containing CP, MPs and CP-MPs (the concentration of cyclophosphamide in each well containing CP and CP-MPs was in accordance with the article [26], where the EC_50_ of CP against 3T3 cells was determined to be 10.8 mmol L^-1^).

For the MTT assay, cell suspension was incubated with the studied samples for 72 hours. Then, the medium was removed, 200 μL of MTT solution in DMEM (0.25 mg mL^-1^) was added to each well and the cells were incubated for 4 h at 37 °C in a CO_2_ incubator. The resulting formazan was dissolved in DMSO, and the absorbance was determined at 550 nm (the reference wavelength was 650 nm) using iMark Microplate Reader (Bio-Rad Laboratories, USA). Metabolic activity (as an indicator of cell viability) was calculated relative to untreated cells according to Equation (2):



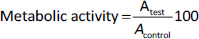

(4)


where *A*_test_ is the absorbance of the test sample and *A*_control_ is the absorbance of the control sample.

Cytotoxicity was studied using the ReadyProbes™ Cell Viability Imaging Kit (Thermo Fisher, USA) to perform double staining: Hoechst 33342 (nuclei of live cells, blue) and SYTOX Green (nuclei of dead cells, green). Images were obtained using Leica DM6000 B TL (BF) +Fluo digital microscope (Leica Microsystems, Germany). The number and percentage of viable cells were analysed using the open-source software ImageJ [27], and cell viability was determined as a percentage of untreated cells.

Each measurement was carried out in triplicate.

## Results and discussion

In the present research both blank and cyclophosphamide-loaded poly(3-hydroxybutyrate-co-3-hydroxyvalerate) microparticles were successfully prepared by spray drying method. The following characteristics of the obtained microparticles were determined: average hydrodynamic diameter, surface morphology, zeta potential, encapsulation efficiency, drug loading, thermal properties and cytotoxicity against 3T3 fibroblast cells.

### Size and size distribution

**Figure 1.**
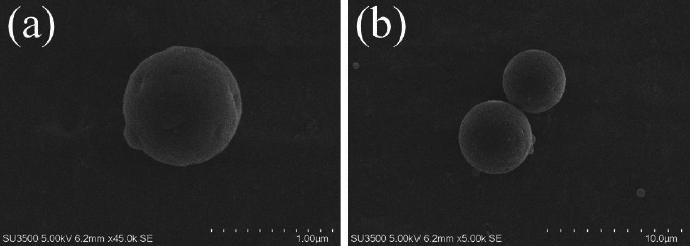
SEM images of the obtained microparticles: (a) MPs. (b) CP-MPs.

The size and size distribution determine the scope of application of microparticles as delivery systems [28-30]. The dimensional characteristics (average hydrodynamic diameter, PDI) of the obtained microparticles were determined using the dynamic light scattering (DLS) method. The average hydrodynamic diameters of MPs and CP-MPs were 1.35±0.02 μm (PDI = 0.12±0.01) and 4.21±0.04 μm (PDI = 0.22±0.02), respectively. The DLS results are consistent with the results of scanning electron microscopy (Figure 1).

In general, an increase in the size of loaded PHBV-based microparticles compared to blank ones is a typical phenomenon, which is confirmed by the research of Dorokhin *et al.* (an increase in the average diameter of microparticles after encapsulation of rifampicin from 1.01 to 2.04 μm was noted) [31], Masood *et al.* (from 0.21 to 0.27 μm; ellipticine encapsulation) [32] and Vidal *et al.* (from 0.61 to 0.66 μm; quercetin encapsulation) [33]. An increase in the size of microparticles after encapsulation is also noted for microparticles based on PHA of other compositions [34-36].

The main routes of administration of CP are oral, intravenous and intramuscular [37]. It is known [38-41] that microparticles with a diameter of 5 to 8 μm are suitable for intramuscular administration (*e.g.* FDA-approved medication Lupron Depot® contains PLGA microparticles of approximately 8 μm in size [42]). Thereby, the obtained micronized form of cyclophosphamide can be suitable for this route of administration.

### Zeta potential

The electrokinetic potential (zeta potential (ZP)) of a microparticle is the difference in the potential of the dispersion medium and the stationary layer of liquid surrounding the particle. The sign and value of the ZP allows to predict the applicability of microparticles in biological systems, as well as their tendency to aggregate. When microparticles are administered into the body, they are recognized as foreign material and are usually removed by the mononuclear phagocyte system [43]. The elimination of microparticles by the mononuclear phagocyte system tends to accelerate with the increase in surface charge [44].

It is considered that the zeta potential value exceeding 30 mV in absolute value is a marker of the aggregative stability of particles [45,46]. Moreover, the ZP of loaded microparticles may indicate the nature of encapsulation of the charged substance [47].

The zeta potentials of the obtained particles were determined to be -35.1±0.3 and -34.2±0.2 mV for MPs and CP-MPs, respectively, which indicates a good aggregative stability of the systems. Similar values of the zeta potentials are noted for various polymer carriers: Corrado *et al.* -35.1±0.3 mV (PHBHHx) [48], Murueva *et al.* -31.8±6.6 mV (P3HB) [49], Lipaikin *et al.* -39.6±0.4 mV (P3HB) [50], Xu *et al.* -31.0±1.4 mV (PLGA) [51], Ruan *et al.* -30.9±0.8 mV (PLA-PEG-PLA) [52].

It was noted that encapsulation of cyclophosphamide did not lead to any significant change in the zeta potential value, which may indicate the formation of microcapsules (the polymer matrix forms a membrane with a reservoir containing the encapsulated substance) or micromatrices (the encapsulated substance and polymer are evenly distributed throughout the volume of the microparticle). The interaction of the encapsulated drug with the surface of microparticles can affect ZP value.

Similar results were obtained by Shershneva *et al.* [53]: the encapsulation of tebuconazole of different concentrations into the P3HB matrix did not change the values of zeta potentials of microparticles. The values of ZPs were in the range from -32.6±0.9 to -35.7±2.0 mV.

### Determination of CP encapsulation efficiency and drug loading

The encapsulation efficiency and the drug loading are the indicators of the success of the encapsulation process [54]. EE and DL values were determined spectrophotometrically.

The encapsulation efficiency and the cyclophosphamide loading into the PHBV-based matrix were 23.3±0.3 and 4.1±0.2 %, respectively. Similar values were noted in [55-57]. The relatively low EE and DL values may be caused by the fairly high crystallinity of PHBV (>60 %) [58,59].

The characteristics of the obtained microparticles are presented in Table 1.

**Table 1.** The characteristics of the obtained microparticles.

Sample	*Y* / %	Hydrodynamic diameter, μm	ZP, mV	EE, %	DL, %	PDI
MPs	31.4±0.4	1.35±0.02	-35.1±0.3	_	_	0.12±0.01
CP-MPs	35.1±0.3	4.21±0.04	-34.2±0.2	23.2±0.3	4.1±0.2	0.22±0.02

EE and DL values vary significantly depending on the carrier used. For instance, the encapsulation of *Artemisia turcomanica* extract into niosomal formulations resulted in an EE of 71.21 %, as reported by Keshtmand *et al.* [60]. Similar EE values (81.43 and 84.28 %) were achieved by Li *et al*. [61] when encapsulating temozolomide and tetra(4-carboxyphenyl)porphyrin in liposomes. Nonetheless, drug encapsulation efficiency in polymeric carriers is generally considerably lower (Salmasi *et al*. [62] encapsulated doxorubicin in PLGA nanoparticles and achieved an EE of 26.66 %).

### Thermal properties

The glass transition temperature, cold crystallization point, melting point and enthalpies of fusion and cold crystallization were determined by differential scanning calorimetry (Table 2) [63,64].

**Table 2.** The results of DSC (second heating for MPs and CP-MPs) and TGA analyses.

Sample	*T*_g_ / °C	*T*_m1_ / °C	Δ*H*_1_ / J g^-1^	*T*_m2_ / °C	Δ*H*_2_ / J g^-1^	*T*_cc_ / °C	Δ*H*_cc_ / J g^-1^	*T*_deg_ / °C
CP	_	49.9	102.6	_	_	_	_	132.1
MPs	-0.78	134.3	13.0	145.5	41.8	57.8	-40.7	276.2
CP-MPs	0.91	132.3	4.1	140.8	32.7	67.3	-37.1	267.7

*T*_g_ - midpoint temperature according to ASTM E1356 and ISO 11357; *T*_m_ and *T*_cc_ - peak temperatures according to ISO 11357,

*T*_deg_ - extrapolated onset temperature according to ISO 11358.

**Figure 2.**
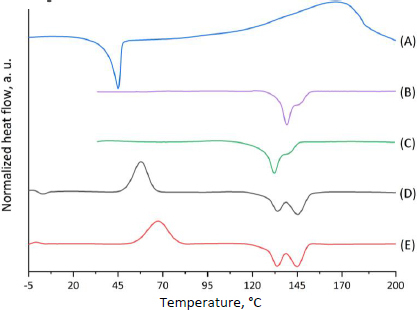
Normalized DSC thermograms of CP (A: 1^st^ heating curve), MPs (B: 1^st^ heating curve), CP-MPs (C: 1^st^ heating curve), MPs (D: 2^nd^ heating curve), CP-MPs (E: 2^nd^ heating curve).

The CP thermogram (Figure 2, curve A) shows a melting peak at 49.9 °C (which corresponds to the literature data [65,66]). Subsequent heating of cyclophosphamide probably leads to degradation or evaporation of CP, as evidenced by a wide peak in the temperature range from 120 to 200 °C.

DSC thermograms for both types of microparticles recorded during the first heating cycle (Figure 2, curves B and C) are similar in shape but differ in the position of the melting peaks. Since the first heating cycle characterizes the initial state of the polymer samples, it is fair to assume that the *T*_m_ shift is caused by the different packing of the polymer chains during the micronization process. As mentioned above, the thermal behaviour of the studied samples is affected by the orientation of the polymers during the micronization process, so it is important to perform the second heating cycle to estimate *T*_g_, *T*_cc_ and *T*_g_. DSC thermograms for both types of microparticles (Figure 2, curves D and E) are similar in shape and have almost the same positions of the melting peaks: 134.3 °C (*T*_m2_ = 145.5 °C) and 132.3 °C (*T*_m2_ = 140.8 °C) for MPs and CP-MPs, respectively. However, a noticeable shift in the position of the cold crystallization peak (57.8 °C for MPs and 67.3 °C for CP-MPs) and an increase in the glass transition temperature from -0.78 °C to 0.91 °C were noted. The changes in *T*_cc_ and *T*_g_ are associated with the effect of CP thermal decomposition products on the thermal properties of the polymer: decomposition of CP (132.1 °C) occurs directly in the polymer melting zone (132.2 °C).

It was noted that there is no melting peak of CP at the CP-MPs thermograms (Figure 2, curves B and C), which may be caused by CP amorphization during its encapsulation into microparticles.

**Figure 3.**
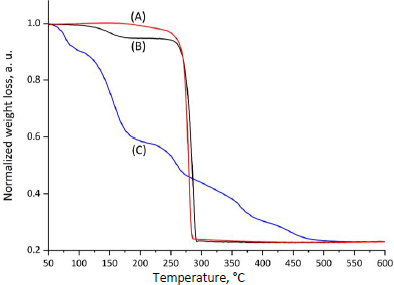
Normalized TGA thermograms of (A) CP-MPs, (B) MPs and (C) CP.

The thermal stability of CP, MPs, and CP-MPs was studied using thermogravimetric analysis. TGA curves and corresponding data for CP, MPs and CP-MPs are shown in Figure 3 and Table 2, respectively.

The TGA thermogram of CP shows several decomposition steps. At the first step (52.5 to 99.0 °C), a loss of 7.9 % of the sample mass (water loss) is noted. The next step (132.1 to 174.5 °C), where the degradation rate reaches its maximum, is characterized by the loss of 25.8 % of mass, consistent with the DSC data. The total mass loss in the remaining areas is 20.7 %.

The thermal degradation of both types of microparticles showed two degradation steps. The first step is probably associated with the loss of residual DCM used in the preparation of microparticles.

The second step is typical for total PHA degradation [33,67]. The degradation temperature of CP-MPs is slightly lower than *T*_deg_ of unloaded microparticles (276.2 and 267.7 °C for MPs and CP-MPs, respectively). Such a decrease in *T*_deg_ of CP-MPs could potentially be caused by the influence of cyclophosphamide degradation products, which, at high temperatures, contribute to the accelerated degradation of the carrier. A decrease in *T*_deg_ of the polymer material was also noted in [68,69] after encapsulation of paclitaxel into the PLGA-PEG matrix and in [70] after encapsulation of metformin into PHBV-based microparticles.

### FTIR spectroscopy

**Figure 4.**
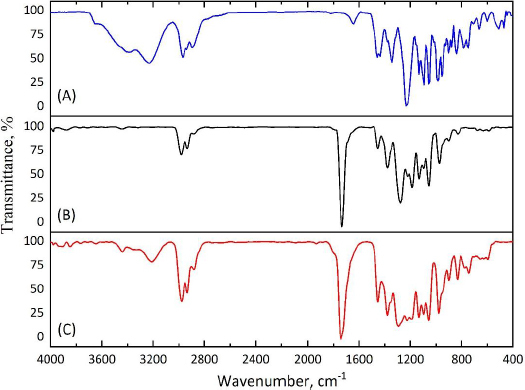
FTIR spectra of (A) CP, (B) MPs and (C) CP-MPs.

To confirm the fact of successful encapsulation of cyclophosphamide into the PHBV matrix, FTIR spectra of pure cyclophosphamide monohydrate and both types of microparticles (MPs, CP-MPs) were obtained (Figure 4). The absence of new absorption bands in the FTIR spectrum of CP-MPs compared to spectra of CP and MPs indicates no chemical interaction between cyclophosphamide and PHBV.

**Figure 5.**
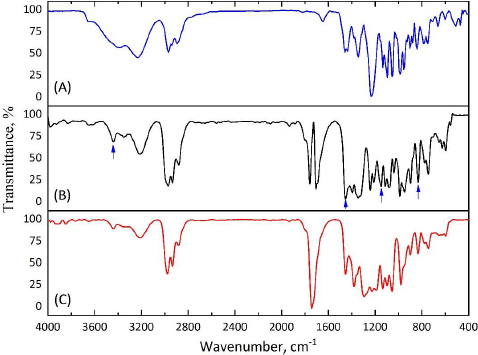
(A) FTIR spectrum of CP, (B) subtracted FTIR spectrum of MPs from CP-MPs and (C) FTIR spectrum of CP-MPs.

The success of encapsulation is evidenced by the appearance of the absorption bands in the FTIR spectrum of CP-MPs corresponding to the structural elements of cyclophosphamide: 3440 cm^-1^ (N-H), 1451 cm^-1^ (P-O-С), 1148 cm^-1^ (P=O), 873 cm^-1^ (CH_2_-Cl) [71]. Due to the low intensity of the corresponding absorption bands, they can be seen in the spectrum obtained by subtracting the matrix spectrum (MPs) from the film spectrum (CP-MPs) (Figure 5).

As a result of encapsulation, cyclophosphamide can be localized both inside microparticles, forming micromatrices or microcapsules, and/or on their surface. The adsorption of CP on the surface of microparticles would probably cause a noticeable change in the zeta potential of CP-MPs, however, the zeta potentials of MPs (-31.4±0.4 mV) and CP-MPs (-35.4±0.3 mV) do not differ significantly.

**Figure 6.**
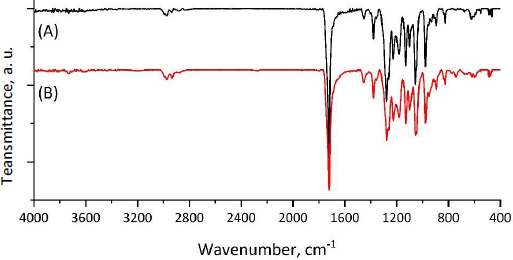
Normalized ATR-FTIR spectra of (A) MPs and (B) CP-MPs.

In order to confirm the absence of changes in the chemical structure of the MPs surface after CP encapsulation, the ATR-FTIR spectra of MPs and CP-MPs were obtained. As can be seen from Figure 6, there are no absorption bands of cyclophosphamide in the ATR-FTIR spectrum of CP-MPs and the positions of the absorption bands for both MPs and CP-MPs are completely identical. Thus, we suppose that CP is predominantly localized inside microparticles.

### In vitro release

**Figure 7.**
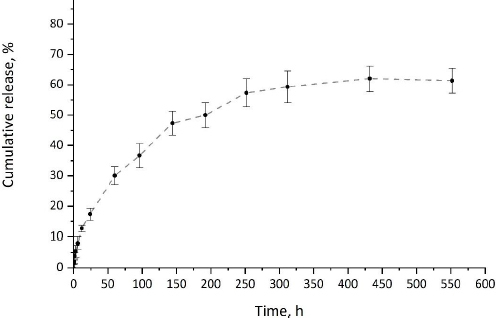
Release profile (kinetic curve) of CP-MPs.

The release of CP from CP-MPs was investigated *in vitro* under physiological conditions (pH 7.4, 37 °C). *In vitro* drug release profile of cyclophosphamide from CP-MPs is presented in Figure 7.

Since PHA-based materials are hydrophobic and are not prone to hydrolytic degradation in the absence of enzymes or acids/bases [72], the dominant mechanism of the release of the encapsulated drug from PHA-MPs is diffusion [73]. The release of cyclophosphamide from CP-MPs has a two-stage pattern typical for systems based on polyhydroxyalkanoates [74,75]. At the initial stage of CP release, a slight burst effect caused by the desorption of cyclophosphamide from the surface of microparticles is observed. During the first 6 hours of exposure, about 12 % of the encapsulated substance is released. At the second stage, a slow release of the drug caused by the diffusion of CP to the surface of the MPs occurs. It was noted that the maximum release of cyclophosphamide (62 %) was achieved in 432 hours (18 days), which correlates with literature data on the duration of cyclophosphamide therapy.

Mathematical models such as zero-order, first-order, Higuchi and Hixson-Crowell were fitted to the *in vitro* release profile [76]. To determine the optimal model, the model with the highest coefficient of determi-nation (*R*^2^) was selected (Table 3) [77]. Based on this criterion, the Higuchi model was found to be best suited for the release kinetics of cyclophosphamide from CP-MPs (*R*^2^ = 0.96), indicating that the release of CP from microparticles is controlled by Fickian diffusion [78].

**Table 3.** *R*^2^ values of various drug release kinetic models.

Kinetic model	Zero-order	First-order	Higuchi	Hixson-Crowell
*R* ^2^	0.79	0.85	0.96	0.83

### In vitro cell viability and cytotoxicity assays

The toxic effect of the micronized form of CP in comparison with unloaded microparticles and pure CP was estimated against fibroblast cell line 3T3.

**Figure 8.**
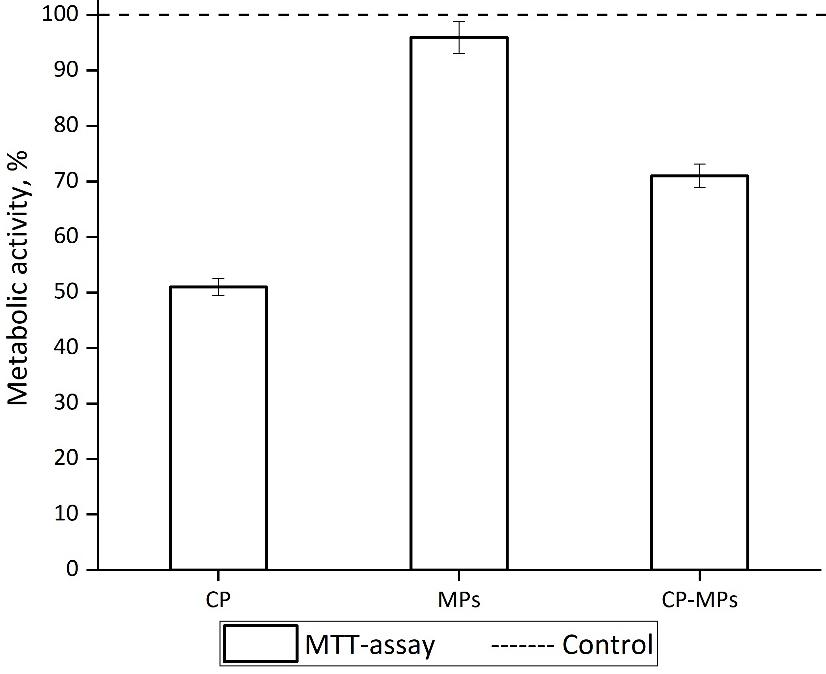
Metabolic activity of 3T3 cells in the presence of CP, MPs and CP-MPs.

According to the results of the MTT assay (Figure 8), the cultivation of fibroblasts with CP and CP-MPs resulted in a decrease in the number of viable cells.

The reduced toxic effect of CP-MPs may be associated with sustained release of the CP from the microparticles (according to the kinetic study, approximately 33 % of encapsulated cyclophosphamide is released over 3 days). The reduced cytotoxicity of microencapsulated forms of drugs is also noted in [79] in the case of encapsulating rifampicin into the PHBV matrix (V79 cells) and 5-fluorouracil into the PLGA matrix (MCF7 cells) [80]. The cultivation of cells with blank microparticles does not lead to a decrease in the metabolic activity of 3T3 in comparison with the untreated control culture.

**Figure 9.**
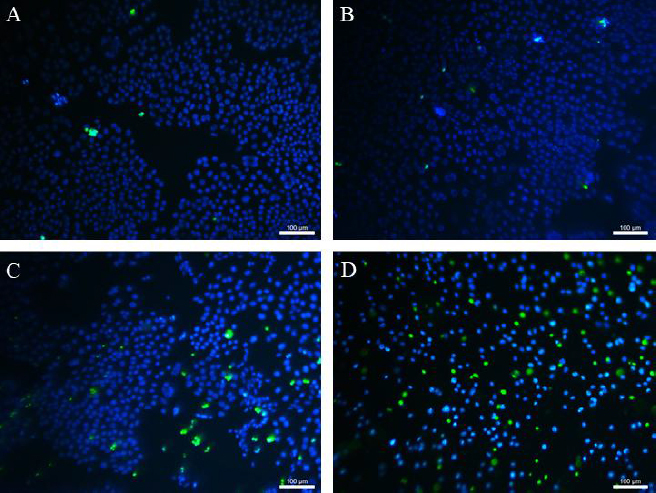
*In vitro* LIVE/DEAD assay: (A) control, (B) MPs. (C) CP-MPs. (D) CP (blue = live cells; green = dead cells).

Figure 9 shows the adhered fibroblasts after staining with Hoechst 33342 and SYTOX Green. Almost none of the dead fibroblasts (green in colour) could be identified during the cultivation with MPs. It means that MPs did not affect the viability of adherent cells compared to untreated control culture. In the case of cultivation 3T3 with CP and CP-MPs, 52 and 24 % of dead cells were found, respectively.

Thus, it was found that microencapsulation reduces the cytotoxicity of cyclophosphamide against healthy cells.

## Conclusions

In this study, cyclophosphamide-loaded poly(3-hydroxybutyrate-co-3-hydroxyvalerate) microparticles were obtained and characterized. It was found that the loading of CP into PHBV microparticles leads to changes in the physical characteristics of the CP-containing formulation compared to the blank microparticles: the decrease of melting points and degradation temperature as well as increase of microparticles diameter, glass transition temperature and cold crystallization temperature were noted. Furthermore, *in vitro* cell viability assay revealed that the encapsulation of cyclophosphamide into PHBV microparticles decreased CP-MPs cytotoxicity against 3T3 cells compared to pure CP. In the future, in order to improve the uptake of the carrier by tumour cells, increase the residence in systemic circulation, facilitate particles' penetration into the interstitial space of the tumour via the EPR effect and circumvent premature drug release, the obtained system can be modified. This can be accomplished by reducing the size of the particles, coating the particles' surfaces with a stealth shell, and functionalizing their surface with some ligands to provide active targeting and prevent macrophage uptake. Overall, this study offers promising prospects for cancer therapy in the future.

Supplementary material Additional data are available at https://pub.iapchem.org/ojs/index.php/admet/article/view/2434, or from the corresponding author on request.
