# Supplementary material for: Spray-dried cyclophosphamide-loaded polyhydroxyalkanoate microparticles: design and characterization
Source: ADMET DMPK. 2024 Oct 9;12(6):925–42. doi: 10.5599/admet.2434 (PMC11661805; doi:10.5599/admet.2434)
Supplement: Supplementary file 1 — Supplementary material [file ADMET-12-2434-S1.pdf]

Open Access : ISSN : 1848-7718

http://www.pub.iapchem.org/ojs/index.php/admet/index

Supplementary material to

Spray-dried cyclophosphamide-loaded polyhydroxyalkanoate microparticles: design and characterization

Sergei Lipaikin[1,*], Aleksei Dorokhin[1], Galina Ryltseva[1], Andrey Oberenko[1], Evgeniy Kiselev[1,2], Alexander Shabanov[3], Tatiana Volova[2] and Ekaterina Shishatskaya[1]

[1]Siberian Federal University, 79 Svobodny pr., Krasnoyarsk 660041, Russia
[2]Institute of Biophysics SB RAS, Federal Research Center "Krasnoyarsk Science Center SB RAS", 50/50 Akademgorodok, Krasnoyarsk 660036, Russia
[3]L.V. Kirensky Institute of Physics, Siberian Branch of the Russian Academy of Sciences, 50/38 Akademgorodok, Krasnoyarsk 660036, Russia

ADMET & DMPK **12(6)** (2024) 925-942; https://doi.org/10.5599/admet.2434

The effect of argon gas flow rate and temperature on the particles' average diameter and zeta potential (Table S1).

A – Argon gas flow rate (A100, A90, A80, A70 for 35.0, 31.5, 28.0 and 24.5 m^3 h^{-1}, respectively).

T – Inlet temperature (T100, T90, T80, T70 for 100, 90, 80 and 70 °C respectively).

According to the results (the lowest zeta potential value and the lowest hydrodynamic diameter) argon gas flow rate and inlet temperature were chosen to be 35.0 m^3 h^{-1} and 100 °C, respectively.

Table S1. The effect of argon gas flow rate and temperature on the particles' average diameter and zeta potential.

	Zeta potential, mV	Average diameter, nm
A100 T70	-33.7	3861
A100 T80	-31.9	4177
A100 T90	-32.8	1931
A100 T100	-35.1	1349
A90 T70	-34.2	2778
A90 T80	-34.3	3348
A90 T90	-32.3	2818
A90 T100	-30.8	2992
A80 T70	-29.9	2217
A80 T80	-30.2	2066
A80 T90	-32.2	4559
A80 T100	-32.5	2031
A70 T70	-33.1	2706
A70 T80	-32.2	2904
A70 T90	-33.7	4273
A70 T100	-35.0	5177
