# Supplementary material for: Spray-dried cyclophosphamide-loaded polyhydroxyalkanoate microparticles: design and characterization
Source: ADMET DMPK. 2024 Oct 9;12(6):925–42. doi: 10.5599/admet.2434 (PMC11661805; doi:10.5599/admet.2434)
Supplement: Supplementary file 2 — Supplementary material [file ADMET-12-2434-S1.docx]

*ADMET & DMPK 12(6) (2020) S17*

*
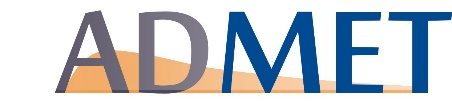
***Open Access : ISSN : 1848-7718**[***http://www.pub.iapchem.org/ojs/index.php/admet/index***](http://www.pub.iapchem.org/ojs/index.php/admet/index)

Supplementary material to

**Spray-dried cyclophosphamide-loaded polyhydroxyalkanoate microparticles: design and characterization**

Sergei Lipaikin^1,^*, Aleksei Dorokhin^1^, Galina Ryltseva^1^, Andrey Oberenko^1^, Evgeniy Kiselev^1,2^, Alexander Shabanov^3^, Tatiana Volova^2^ and Ekaterina Shishatskaya^1^

*^1^**Siberian Federal University, 79 Svobodny pr., Krasnoyarsk 660041, Russia
^2^Institute of Biophysics SB RAS, Federal Research Center “Krasnoyarsk Science Center SB RAS”, 50/50 Akademgorodok, Krasnoyarsk 660036, Russia
^3^L.V. Kirensky Institute of Physics, Siberian Branch of the Russian Academy of Sciences, 50/38 Akademgorodok, Krasnoyarsk 660036, Russia*

ADMET & DMPK **12(6)** (2024) 925-942; <https://doi.org/10.5599/admet.2434>

The effect of argon gas flow rate and temperature on the particles’ average diameter and zeta potential (Table S1).

*A* – Argon gas flow rate (A100, A90, A80, A70 for 35.0, 31.5, 28.0 and 24.5 m^3^ h^-1^, respectively).

*T* – Inlet temperature (T100, T90, T80, T70 for 100, 90, 80 and 70 °C respectively).

According to the results (the lowest zeta potential value and the lowest hydrodynamic diameter) argon gas flow rate and inlet temperature were chosen to be 35.0 m^3^ h^-1^ and 100 °C, respectively.

**Table S1.** The effect of argon gas flow rate and temperature on the particles’ average diameter and zeta potential.

|  | Zeta potential, mV | Average diameter*,* nm |
| --- | --- | --- |
| A100 T70 | -33.7 | 3861 |
| A100 T80 | -31.9 | 4177 |
| A100 T90 | -32.8 | 1931 |
| A100 T100 | -35.1 | 1349 |
| A90 T70 | -34.2 | 2778 |
| A90 T80 | -34.3 | 3348 |
| A90 T90 | -32.3 | 2818 |
| A90 T100 | -30.8 | 2992 |
| A80 T70 | -29.9 | 2217 |
| A80 T80 | -30.2 | 2066 |
| A80 T90 | -32.2 | 4559 |
| A80 T100 | -32.5 | 2031 |
| A70 T70 | -33.1 | 2706 |
| A70 T80 | -32.2 | 2904 |
| A70 T90 | -33.7 | 4273 |
| A70 T100 | -35.0 | 5177 |
